# Secondary Data Analyses of Conclusions Drawn by the Program Implementers of a Positive Youth Development Program in Hong Kong

**DOI:** 10.1100/tsw.2010.1

**Published:** 2010-02-12

**Authors:** Andrew M. H. Siu, Daniel T.L. Shek

**Affiliations:** ^1^ Department of Rehabilitation Sciences, The Hong Kong Polytechnic University, Hong Kong; ^2^ Department of Applied Social Studies, The Hong Kong Polytechnic University, Hong Kong; ^3^ Kiang Wu Nursing College of Macau, Macao

**Keywords:** >subjective outcome evaluation, positive youth development, secondary data analysis

## Abstract

The Tier 2 Program of the Project P.A.T.H.S. (Positive Adolescent Training through Holistic Social Programmes) is designed for adolescents with significant psychosocial needs, and its various programs are designed and implemented by social workers (program implementers) for specific student groups in different schools. Using subjective outcome evaluation data collected from the program participants (Form C) at 207 schools, the program implementers were asked to aggregate data and write down five conclusions (n = 1,035) in their evaluation reports. The conclusions stated in the evaluation reports were further analyzed via secondary data analyses in this study. Results showed that the participants regarded the Tier 2 Program as a success, and was effective in enhancing self-understanding, interpersonal skills, and self-management. They liked the experiential learning approach and activities that are novel, interesting, diversified, adventure-based, and outdoor in nature. They also liked instructors who were friendly, supportive, well-prepared, and able to bring challenges and give positive recognition. Most of the difficulties encountered in running the programs were related to time constraints, clashes with other activities, and motivation of participants. Consistent with the previous evaluation findings, the present study suggests that the Tier 2 Program was well received by the participants and that it was beneficial to the development of the program participants.

## INTRODUCTION

The Positive Adolescent Training through Holistic Social Programmes (Project P.A.T.H.S.) is a two-tier program that aims to promote positive youth development in junior secondary school students in Hong Kong[1,2]. The Tier 1 Program consists of a curriculum designed to provide 20 h of training to Secondary 1 to 3 students of participating schools. The Tier 2 Program is a collection of specific intervention programs designed by social service agencies for students with significant psychosocial needs in different schools. To identify students suitable for the Tier 2 Program, the school and the social service agency involved in conducting the program review the academic and behavioral records of students, and teachers' ratings or recommendations. The target group, students with significant psychosocial needs, refers to those who experienced intense conflicts with parents or family, had difficulties in school adjustment and peer relationships, or had increased risk for mental health problems. About one-fifth of the students at a school enrolled in the Tier 1 Program would normally be selected to join the Tier 2 Program.

The program implementers, who are mostly social workers, identify the needs of the selected students and design appropriate programs for them. The type of programs that are commonly conducted include (1) mentorship programs, (2) mental health promotion, (3) adventure-based counseling, (4) parenting programs, (5) service learning programs, and (6) resilience enhancement programs. Because of the diversity of programs across different schools, it is not feasible to develop an experimental or standardized measurement of outcomes of the Tier 2 Program as a whole. The Tier 2 Program was evaluated by eliciting the views of participants, program implementers, instructors, and participants (students and sometimes parents/caregivers). The respondents complete a Subjective Outcome Evaluation questionnaire (Form C) that gauges their degree of satisfaction with the program, perceived benefits, difficulties they encountered, and their recommendations for improvement[1].

In some previous studies on the Tier 2 Program of the Project P.A.T.H.S. at the Secondary 1 level (7$^{th}$ grade), respondents were found to have very positive perceptions of the program, the instructors, and the program benefits[3,4]. As the Tier 2 Program has now been extended to Secondary 2 students in the Experimental Implementation Phase, there is a need to examine Secondary 2 students' perceptions of the Tier 2 Program.

Most Tier 2 programs are tailored to meet the specific needs of students in a particular school, and there is a wide variety of program content and approaches among schools. As a general requirement of Tier 2, social service agencies need to design programs with reference to the 15 positive youth development constructs covered in the Project P.A.T.H.S., in addition to the specific objectives of the program. A survey of the current objectives and content of Tier 2 programs showed that they often focus on several developmental constructs of the Project P.A.T.H.S., including the development of self-concept, behavioral competence, cognitive competence, social competence, and prosocial involvement. Many programs adopt experiential learning as the key approach. An adventure-based counseling approach, and volunteer training and services, were also very popular[3,4].

Experiential learning is learning by doing and by reflection. The experiential learning approach is widely used in primary and secondary school education, in adult education, and in management training, and is particularly suitable for programs that aim to increase self-understanding and interpersonal effectiveness. Experiential learning aims to help participants to derive meaning from learning experiences, often in the form of groupwork[5]. Kolb presented a four-step cycle of experiential learning: concrete experience, reflective observation, abstract conceptualization, and active experimentation[6]. The cycle could happen in this sequence or in nearly any other sequence. The key criteria for designing experiential learning activities are that the activities need to have personal significance and meaning for the student, that the students need to be personally engaged (in terms of their senses, thoughts, feelings, and personality), and that the activities should involve continuous reflection[6]. Instructors need to establish a sense of trust, respect, openness, and concern for the well-being of students, and recognize the prior learning that they have brought with them into the new learning environment. While the experiential learning model is widely adopted in education and training, there is also some strong criticism of its basic tenets (what exactly is "experiential learning"?), of the insufficient attention it pays to the process of reflection (on one's feelings), of its application of the learning style inventory, and of the lack of systematic research efforts into it[7,8].

Developed under the experiential learning model, adventure-based counseling (ABC) engages participants in challenging and adventurous activities, and aims to foster personal qualities like courage, resilience, responsibility, belonging, mastery, autonomy, and altruism[9,10]. The experiential activities in ABC also serve as metaphors for real-life issues, and it is postulated that participants who successfully overcome the challenges in the program could transfer their experiences to other life challenges in the present or future[11]. The ABC approach has been widely used with adolescents, and in particular with delinquent youth, and there is some evidence that the approach could improve self-esteem and coping skills of young people[11,12]. However, there are also critics who have questioned the research evidence of the ABC programs, and how far the outcomes of these programs can be generalized and sustained in real-life situations.

Participation in volunteer training and services is the second approach that is commonly used in Tier 2 programs. The engagement of young people in volunteering provides an excellent platform for personal development, socialization of prosocial norms and behavior, recognition for positive behavior, and leadership training. Volunteer training requires different levels of student involvement and competence, and the level of participation and training is flexible. While volunteer work often benefits the person receiving the service, there is evidence that the volunteers also benefit from the process of providing help to others. Some studies have shown that young persons who participate in volunteer work have increased levels of empathy and altruism, increased self-efficacy, and fewer problem behaviors[13,14]. Through volunteer work, young people obtain real-life opportunities to practice their interpersonal and organizing skills, and to establish bonds with healthy people and learn from models or mentors[15].

There are many factors that influence the implementation and effectiveness of experiential learning programs. These factors include the characteristics of the program, the instructors, and the teaching-learning process. First, program characteristics such as its relevance to the needs of students, the training mode, and support from participants are important[16,17]. Second, the quality and leadership style of instructors are important determinants of the success of programs. In particular, participants often appreciate good rapport, a caring and responsible attitude, professional teaching skills, a good presentation style, and the ability to arouse their interests and involvement[18]. Third, successful programs are characterized by good instructor-student interaction, high levels of peer interaction, and high levels of peer involvement[19].

This study examined the subjective outcomes of the Tier 2 Program by eliciting the views of participants and program implementers on the need and relevance of the Tier 2 programs, on the quality of instruction, on program effectiveness, on the difficulties of program implementation, and on how to improve the programs. The results can provide information about the quality of programs, as well as suggestions about how to improve future programs. In addition, as documentation of practice research is rare in the social work literature in Hong Kong[20], this paper describes a systematic approach in documenting the effectiveness of youth programs designed by social service agencies.

## METHODS

The objectives of this study were to examine the overall effectiveness of the Tier 2 Program of the Project P.A.T.H.S. The subjective outcomes of Tier 2 programs perceived by participants were collected using Form C from more than 10,000 participants of 200 schools in an earlier qualitative evaluation study. In this study, program implementers (often social workers) conducted a data aggregation of data in Form C according to research guidelines, producing summaries of qualitative comments. These summaries of qualitative data form the dataset for the secondary analysis in this study. A secondary analysis of the conclusions drawn from these summaries is expected to provide an overall picture of the effectiveness of the Tier 2 Program in five aspects: (1) participants' view of the program, (2) views about instructors, (3) perceived program effectiveness, (4) difficulties encountered, and (5) recommendation for the program.

### Dataset for Secondary Data Analyses

In the 2006/07 school year, 207 schools joined the Tier 1 Program of the Project P.A.T.H.S. in the Experimental Implementation Phase. Through the review of student needs by teachers and parents, and/or the results of screening questionnaires, a total of 12,092 Secondary 2 students at these schools were invited and agreed to participate in the Tier 2 Program. A total of 1,102 parents and teachers of these students also participated in the Tier 2 Program. Thus, the total number of participants in the program was 13,194. The mean number of participants joining the Tier 2 Program per school was 63.74 (range: 14–308). The average number of sessions (normally lasting 1.5–3 h) provided per school was 22.91 (range: 6–62). On completion of the programs, a total of 10,255 participants (mean = 49.54 participants per school, range: 6–294) completed the Subjective Outcome Evaluation Form (Form C)[1]. The overall response rate was 77.72%. All participants provided written consent to join the study when they enrolled in the Tier 2 Program.

The Subjective Outcome Evaluation Form (Form C) was designed by Shek and Siu[1] with the aim of measuring participants' perceptions of the Tier 2 Program. It was developed through a content review of evaluation forms that are currently used by social services agencies for assessing participants' satisfaction with personal growth programs. There are seven parts in this evaluation form:

1. Participants' perceptions of the program, such as their perceptions of program arrangement, quality of service, appropriateness of the program, and interaction among the participants (eight items).
2. Participants' perceptions of the instructors, such as their perceptions of how far the instructors are well prepared in conducting the program, the instructors' professional attitude and knowledge, and the instructors' interaction with the participants (eight items).
3. Participants' perceptions of the effectiveness of the program, such as their perceptions of the promotion of problem-solving skills, behavioral modification, and positive change (eight items).
4. Things that the participants appreciated the most (open-ended question).
5. The participants' opinion of the instructors (open-ended question).
6. Things that the participants learned from the program (open-ended question).
7. Areas that require improvement (open-ended question).

To facilitate the process of eliciting subjective evaluations of the Tier 2 programs, guidelines for data collection and analysis were listed in a standardized manual. Based on the manual, training was provided to the program implementers during 20-h training workshops on how to collect and analyze the data using Form C. A responsible worker in each school was required to complete an Evaluation Report, where the quantitative and qualitative findings from Form C were summarized and described. In the last section of the report, the social worker preparing the report for each school was requested to write down five conclusions about the program and its effectiveness that could give an overall picture of the perceived effectiveness of the Tier 2 Program. This paper examines these five conclusions drawn by the program implementers.

## Data Analyses

The data were analyzed using content analyses techniques[3] by two research assistants. There were three steps in the data analysis process. First, raw codes were developed for words, phrases, and sentences that formed meaningful units in each conclusion at the raw response level. Second, the codes were further combined to reflect higher-order attributes at the category of codes level. For example, the response "the program is helpful to students" at the raw response level could be subsumed under the category "general program benefit", which could be further subsumed under the broad theme of "benefits of the program" (see Table 1).

Since the authors designed the Project P.A.T.H.S. program, they were conscious of their own biases and their expectation of the program to be effective. As such, they were not directly involved in the data analyses. In addition, in order to minimize potential bias, both intra- and inter-rater reliability on the coding was calculated. For intrarater reliability, the research assistant primarily responsible for coding coded

**TABLE 1**
**Participants' Views on the Program**

| Category | Response | Nature of the Response | | | | Total |
|---|---|---|---|---|---|---|
| | | Positive | Neutral | Negative | Undecided | |
| Satisfaction level | Liked the program | 4 | | | | 4 |
| | Satisfied with the program | 41 | | | | 41 |
| | Positive views about the program | 15 | | | | 15 |
| | Satisfied with the arrangement | 18 | | | | 18 |
| | The program is meaningful | 3 | | | | 3 |
| | The program is able to achieve its goals | 19 | | | | 19 |
| | High attendance rate | 6 | | | | 6 |
| | Students' active participation | 25 | | | | 25 |
| | Parents support the program | 4 | | | | 4 |
| | Worth continuing | 4 | | | | 4 |
| | Would join the program again | 11 | | | | 11 |
| | Would recommend the program to others | 8 | | | | 8 |
| | Positive comments | 5 | | | | 5 |
| | Neutral comments | | 5 | | | 5 |
| | **Subtotal** | **163** | **5** | **0** | **0** | **168** |
| Program content | Comprehensive content | 6 | | | | 6 |
| | Meets students' needs | 34 | | | | 34 |
| | Provides holistic care to students | 2 | | | | 2 |
| | Provides positive experiences to students | 5 | | | | 5 |
| | Provides a platform for students to interact | 5 | | | | 5 |
| | Liked and are satisfied with the activities | 43 | | | | 43 |
| | Diversified activities | 9 | | | | 9 |
| | Benefit from the experiential learning | 10 | | | | 10 |
| | Students' improvements are recognized by others | 10 | | | | 10 |
| | Other positive comments | 31 | | | | 31 |
| | Neutral comments | | 6 | | | 6 |
| | Content can be more comprehensive and interesting | | 2 | | | 2 |
| | Content and activities are unattractive to students | | | 3 | | 3 |
| | Cannot meet students' needs | | | 3 | | 3 |
| | Other negative views | | | 3 | | 3 |
| | **Subtotal** | **155** | **8** | **9** | **0** | **172** |
| Other | Good atmosphere | 4 | | | | 4 |
| | Able to complete the whole program | 2 | | | | 2 |
| | Schools' cooperation | 7 | | | | 7 |
| | Other | 2 | | | | 2 |
| | **Subtotal** | **15** | **0** | **0** | **0** | **15** |
| **Total responses** | | **333** | **13** | **9** | **0** | **355** |

20 randomly selected responses without looking at the original codes given. This set of codes was then compared with coding done when the assistant was allowed to refer to the code book. For inter-rater reliability, another research assistant coded 20 randomly selected responses without knowing the original codes given at the end of the scoring process. Reliability between the two assistants was estimated.

Following the principles of qualitative analysis proposed by Shek et al.[21], the following attributes of the study regarding data collection and analysis were highlighted. First, a general qualitative orientation was adopted. Second, the sources of data (e.g., number of participants) for the analyses were described. Third, the issues of bias and ideological preoccupation were addressed. Fourth, inter- and intrarater reliability information was presented. Fifth, the categorized data were kept in a systematic filing system in order to ensure that the findings were auditable. Finally, possible explanations, including alternative explanations, were considered.

## RESULTS

From the 1,035 conclusions in the 207 reports, 1,640 meaningful units were extracted. These raw responses were further categorized into several categories, and summarized in five tables, including views of the participants on the program (Table 1), views on the instructors (Table 2), perceived effectiveness of the program (Table 3), difficulties encountered (Table 4), and suggestions for improvement (Table 5). Regarding the conclusions related to the participants' perceptions of the program (Table 1), the results showed that most of the responses in the areas of satisfaction and program content were positive. The questionnaire requested participants to give two comments on the program; the average number of comments given per participant was 1.7. Of the 355 responses, 333 were classified as positive (93.80%). The intrarater agreement percentage was 95% and the inter-rater agreement percentage was 90%.

With regard to the view on the instructors of the program (Table 2), most (99.01%) of the responses were positive. In the areas of general and specific appreciation of the instructors, all the responses were positive, and half of the responses were positive in the area of "other" appreciation of the instructors. The intrarater agreement percentage was 100% and the inter-rater agreement percentage was 95%.

The responses related to perceived effectiveness of the program are shown in Table 3. There were a total of 911 meaningful units that could be categorized in several levels, including the societal level, the familial level, the interpersonal level (general interpersonal competence and specific interpersonal competence), and the personal level (positive self-image, ways to face adversity, reflection, goal setting, behavioral competence, cognitive competence, and experiences/exploration). All 217 responses were positive (100%). The intrarater agreement percentage was 95% and the inter-rater agreement percentage was 90%.

Table 4 shows the responses related to difficulties encountered in the implementation of the program. Most (81.2%) of the difficulties were related to implementation of programs. Time constraints, attendance, clashes with other school activities, and students' performance were the most commonly mentioned difficulties in implementation. The intrarater agreement percentage was 85% and the inter-rater agreement percentage was 90%.

Table 5 tabulates the responses on principles of good practice and recommendations for the program. The recommendations were largely categorized into those related to the content (n = 63, 33.0%) or implementation of program (n = 44.0%). In terms of program contents, the participants liked experiential learning and adventure-based activities. Outdoors activities that are interesting, challenging, and diversified were most welcome. In terms of implementation, the participants indicated that good relationship with students, support from school, and the arrangement of some award/recognition were important. Quite a substantial number of participants also recommended better program arrangement and increase in the length of the program., The intrarater agreement percentage was 75% and the inter-rater agreement percentage was 80%.

**TABLE 2**
**Responses to the Views about Instructors**

| Category | Response | Nature of the Response | | | | Total |
|---|---|---|---|---|---|---|
| | | Positive | Neutral | Negative | Undecided | |
| General appreciation | Positive views about instructors | 10 | — | — | — | 10 |
| | Appreciate attitude | 34 | — | — | — | 34 |
| | Appreciate performance | 54 | — | — | — | 54 |
| | **Subtotal** | **98** | **0** | **0** | **0** | **98** |
| Specific appreciation | Satisfied with the instructors' delivery strategies | 9 | — | — | — | 9 |
| | Satisfied with the instructors' arrangements | 6 | — | — | — | 6 |
| | Well prepared for the program | 13 | — | — | — | 13 |
| | Built a good relationship with students | 11 | — | — | — | 11 |
| | Understood students | 9 | — | — | — | 9 |
| | Made adjustments to the program continuously | 3 | — | — | — | 3 |
| | Devoted | 4 | — | — | — | 4 |
| | Professional | 30 | — | — | — | 30 |
| | Cared about students | 9 | — | — | — | 9 |
| | Attitude and performance enhanced students' learning | 4 | — | — | — | 4 |
| | **Subtotal** | **98** | **0** | **0** | **0** | **98** |
| Other | Other | 4 | — | — | — | 4 |
| | Instructors satisfied with their own performance | 1 | — | — | — | 1 |
| | Negative comments | — | — | 2 | — | 2 |
| | **Subtotal** | **5** | **0** | **2** | **0** | **7** |
| **Total responses** | | **201** | **0** | **2** | **0** | **203** |

## DISCUSSION

The participants were generally very positive about the Tier 2 Program. While most of the comments were quite general, the specific comments expressed were mainly related to perceived needs and outcomes, and the experiential aspects of the program. The participants largely agreed that the programs were meeting students' needs and that students' improvements were recognized by others. The participants also appreciated the experiential and adventure-based nature of the activities, which may better meet the needs of this group of students than classroom activities.

Not many comments were given about the instructors. Although asked to give two comments about the instructors, an average of only 0.9 comments per respondent (program implementer) was given. While about half of the comments were general comments (such as about the attitude or performance of the instructors), the other half covered three specific areas: (1) the professionalism, attitude, and devotion of the instructors; (2) the good relationships they had with students, and the understanding and care they showed them; and (3) the arrangement and delivery of the programs. In general, the respondents were less inclined to make comments about people (instructors) than about their satisfaction with the program or its perceived benefits. When the participants commented on the instructors, they tended to focus more on

**TABLE 3**
**Responses to Perceived Program Effectiveness**

| Category | Subcategory | Response | Nature of the Response | | | | Total |
|---|---|---|---|---|---|---|---|
| | | | Positive | Neutral | Negative | Undecided | |
| Societal level | - | Enhanced social responsibility and participation | 27 | | | | 27 |
| | | **Subtotal** | **27** | **0** | **0** | **0** | **27** |
| Familial level | - | Strengthened family bonds | 14 | | | | 14 |
| | | **Subtotal** | **14** | **0** | **0** | **0** | **14** |
| Interpersonal level | General interpersonal competence | Improved interpersonal relationships | 71 | | | | 71 |
| | | Enhanced instructors' and students' relationships | 20 | | | | 20 |
| | | Enhanced the relationships with senior form students | 3 | | | | 3 |
| | | **Subtotal** | **94** | **0** | **0** | **0** | **94** |
| | Specific interpersonal competence | Improved communication/social skills | 67 | | | | 67 |
| | | Learned to cooperate with others | 73 | | | | 73 |
| | | Mutual support/trust/appreciation | 23 | | | | 23 |
| | | Appreciated/respected others | 10 | | | | 10 |
| | | **Subtotal** | **173** | **0** | **0** | **0** | **173** |
| Personal level | Positive self-image | Enhanced students' development | 86 | | | | 86 |
| | | Positive impacts on students | 75 | | | | 75 |
| | | Developed clear and positive identity | 16 | | | | 16 |
| | | Enhanced self-understanding | 34 | | | | 34 |
| | | Enhanced self-efficacy | 37 | | | | 37 |
| | | Enhanced self-confidence | 57 | | | | 57 |
| | | Enhanced self-esteem | 17 | | | | 17 |
| | | Enhanced emotional management | 15 | | | | 15 |
| | | **Subtotal** | **337** | **0** | **0** | **0** | **337** |
| | Ways to face adversity | Cultivation of resilience | 27 | | | | 27 |
| | | Enhanced problem-solving skills | 49 | | | | 49 |
| | | **Subtotal** | **76** | **0** | **0** | **0** | **76** |
| | Reflection | Enhanced self-reflection | 16 | | | | 16 |
| | | **Subtotal** | **16** | **0** | **0** | **0** | **16** |
| | Goal setting | Promoted beliefs in the future | 8 | | | | 8 |
| | | Goal setting | 7 | | | | 7 |
| | | **Subtotal** | **15** | **0** | **0** | **0** | **15** |
| | Behavioral competence | Promoted self-management/self-determination | 17 | | | | 17 |
| | | Positive impacts on behavior | 15 | | | | 15 |
| | | Enhanced learning motivation/skills | 12 | | | | 12 |
| | | Enhanced persistence | 7 | | | | 7 |
| | | Learned to be self-disciplined | 16 | | | | 16 |
| | | **Subtotal** | **67** | **0** | **0** | **0** | **67** |
| | Cognitive competence | Enhanced organizing and analyzing abilities | 4 | | | | 4 |
| | | **Subtotal** | **4** | **0** | **0** | **0** | **4** |
| | Experience/ Exploration | Explored/developed potential | 17 | | | | 17 |
| | | Gained successful experience | 14 | | | | 14 |
| | | Provided other learning experiences | 14 | | | | 14 |
| | | **Subtotal** | **45** | **0** | **0** | **0** | **45** |
| Other | — | Benefits to instructors | 3 | | | | 3 |
| | | Benefits to parents | 11 | | | | 11 |
| | | Strengthened the bonding to school | 19 | | | | 19 |
| | | Positive | 10 | | | | 10 |
| | | **Subtotal** | **43** | | | | **43** |
| **Total responses** | | | **911** | **0** | **0** | **0** | **911** |

**TABLE 4**
**Responses to Encountered Difficulties**

| Category | Response | Total |
|---|---|---|
| Difficulties in handling program content | The program is too intensive | 3 |
| | Loose connection between sessions | 2 |
| | Lack of concrete goals in the program | 1 |
| | Students cannot integrate the learned skills and knowledge into daily life | 2 |
| | **Subtotal** | **8** |
| Difficulties in time management and in program implementation | Insufficient evaluation design | 2 |
| | Student-instructor ratio | 5 |
| | Time constraints | 10 |
| | Classroom management | 1 |
| | Difficulty in cultivating a favorable atmosphere | 2 |
| | Difficulty in building relationships with students | 3 |
| | Spend too much time on preparation | 2 |
| | Attendance rate | 11 |
| | Enrollment | 3 |
| | Students' performance | 10 |
| | Clash with other school activities | 13 |
| | Resistance from parents | 5 |
| | Other | 2 |
| | **Subtotal** | **69** |
| Other | Other | 8 |
| | **Subtotal** | **8** |
| **Total responses** | | **85** |

personal qualities (such as the instructors' professionalism) than on the delivery of the programs. This may imply that the participants appreciated the use of self (personal qualities) as the most important tool when working with this group of students. It may also suggest that the program implementers (some are also the instructors) felt it embarrassing to comment on their own performance, or tried to downplay the importance of their own contributions to the success of the Tier 2 programs. There is much evidence that Chinese people tend to report a more egalitarian distribution of contributions to success in group projects than do people from more individualistic cultures[22].

The participants commented very enthusiastically about the perceived effectiveness of the programs. The main perceived benefits of the program were on the individual level, such as that they enhanced self-identity, improved interpersonal and behavioral competence, and the ability to face adversity. Many programs were seen as helpful in improving self-understanding, exploring and developing identity, and enhancing self-concept. This is in line with the key developmental tasks of searching for one's identity, improving one's self-understanding, and building self-efficacy and confidence during adolescence[23]. In addition, the programs were perceived to have a great impact on interpersonal communication and relationships, such as by helping participants to improve social skills and to learn to respect, trust, and appreciation of others. Both interpersonal relationships and self-concept are strongly linked to the emotional development of students, and these few topics may together form an indispensable part of Tier 2 programs. Lastly, many participants commented that the programs were able to help them develop self-discipline, problem-solving skills, self-management skills, persistence, and resilience. In a societal environment in

**TABLE 5**
**Responses to the Recommendations for the Program**

| Category | Subcategory | Response | Total |
|---|---|---|---|
| Good practice | Content | Experiential learning is an effective means | 5 |
| | | Adventure-based activities enhance students' participation | 6 |
| | | Using outdoor activities | 7 |
| | | Interesting/challenging/diversified activities | 4 |
| | | **Subtotal** | **22** |
| | Implementation | Good relationships with students | 10 |
| | | Support from school | 11 |
| | | Discuss with teachers | 7 |
| | | Enrollment strategy | 7 |
| | | Group strategy | 2 |
| | | Harmonious atmosphere | 5 |
| | | Award/prize/gift/recognition | 8 |
| | | **Subtotal** | **50** |
| | Other | Other | 27 |
| | | **Subtotal** | **27** |
| Recommendations | Content | Make fine adjustments to meet the needs of students | 7 |
| | | More interesting/challenging activities | 7 |
| | | More games | 3 |
| | | More outdoors activities | 10 |
| | | Diversified activities | 6 |
| | | A bigger platform for students' interaction | 3 |
| | | Encourage parents' participation | 5 |
| | | **Subtotal** | **41** |
| | Implementation | Reduce the number of sessions | 2 |
| | | Better program arrangement | 12 |
| | | Implement the program at weekends | 2 |
| | | Better cooperation with schools | 3 |
| | | Increase the number of sessions | 13 |
| | | More free time | 2 |
| | | **Subtotal** | **34** |
| | Other | Other recommendations | 17 |
| | | **Subtotal** | **17** |
| **Total responses** | | | **191** |

which so much information competes for our attention, young people need to learn to stay focused in order to be successful[24].

There are several possible reasons why the three key perceived developmental outcomes (self-concept, interpersonal relationships, and self-management) of the programs identified in this analysis emerged. They could be what the participants want to or expect to see in programs, or these perceived benefits could be the objectives of the programs. These benefits may also have emerged because they are more observable than covert changes in emotional, cognitive, and moral development. Also, there were only a few comments suggesting that the programs had an impact at the familial and societal levels, and this may reflect that the programs focused on the individual rather than on the family or the society. Most programs were designed for individuals or small groups, and did not involve parents or teachers. Most programs did not try to address family issues, participation, or adjustment to the community. While this can be regarded as a limitation of the program, it can be argued that the scope of a positive youth development program cannot be too broad. After all, a positive youth development program should not be regarded as a panacea for all adolescent developmental problems.

Many of the difficulties encountered in the program were related to implementation and time constraints. Only a few comments were concerned with the design of the program or its carry-over effects. The reported difficulties were quite similar to the difficulties raised about the implementation of extracurricular activities for the Tier 1 Program of the Project P.A.T.H.S., such as time constraints, clashes with other activities, or difficulties in motivating students to participate.

In the recommendations section, the participants suggested that an experiential, adventure-based program using a diversity of activities would be most beneficial to the students in the Tier 2 Program. Activities need to be able to stimulate interests, be conducted outdoors if possible, involve some significant challenge, and offer recognition of participation. Good rapport with students and good support from the school are essential factors for the success of these programs. In general, these recommendations or principles of good practice were consistent with the other responses to the questionnaire.

In summary, the results from this study showed that the participants perceived that an experiential learning approach is most suitable for organizing Tier 2 programs. The experiential activities should use a wide variety of forms and be of interest to students. The amount of outdoors (or outside school) activities should be increased, and the activities should challenge participants as well as recognize their participation. The key difficulties encountered in running the programs were operational (such as time constraints and clashes with other activities) or concerned how to adequately enhance the motivation and participation of students.

Most participants agreed that the programs were effective, and they observed that the key benefits of the programs were at the individual level rather than at the family or societal level. The major impact of Tier 2 programs were in the areas of self-understanding, self-exploration, self-concept, social skills and interpersonal relationships, and self-management. The participants were generally very satisfied with the Tier 2 Program, and appreciated that the programs could meet the psychosocial needs of students, and that they were generally well designed and suitably tailored for the students.

It should be noted that the data used in this study were conclusions about the program drawn by program implementers. While program implementers are provided with training to summarize the subjective evaluation forms (Form C) completed by participants, it is possible that they will still be biased about the benefits and process of the programs. The value of this study lies more in the way it has highlighted the factors related to success of the Tier 2 programs than in its provision of an index of satisfaction or an evaluation of the outcomes of the program.

In this study, secondary data analyses based on conclusions drawn by the program implementers were analyzed. There are several advantages in asking the program implementers to draw conclusions. First, as they were the coordinator and manager of these programs, they should take part in the evaluation process. Second, by involving the program implementers, their different experiences could be systematically documented. In fact, if the program implementers were not satisfied with the program, they could make use of this opportunity to voice their dissatisfaction. Finally, the approach adopted in this study can enable the researchers to see the program through the eyes of the program implementers, which can strengthen the ecological validity of the evaluation findings.

## ACKNOWLEDGMENTS

The preparation for this paper and the Project P.A.T.H.S. were financially supported by The Hong Kong Jockey Club Charities Trust.
